# Examining heterogeneity of stromal cells in tumor microenvironment based on pan-cancer single-cell RNA sequencing data

**DOI:** 10.20892/j.issn.2095-3941.2020.0762

**Published:** 2022-01-15

**Authors:** Wenhui Wang, Li Wang, Junjun She, Jun Zhu

**Affiliations:** 1 Department of Genetics and Genomic Sciences, Icahn School of Medicine at Mount Sinai, New York, NY 10029, USA; 2 Sema4, a Mount Sinai venture, Stamford, CT 06902, USA; 3 First Affiliate Hospital of Xi’an Jiaotong University, Xi’an 710061, China; 4 Tisch Cancer Institute, Icahn School of Medicine at Mount Sinai, New York, NY 10029, USA

**Keywords:** Stromal cells, tumor microenvironment, pan-cancer single-cell RNA sequencing data

## Abstract

Tumor tissues contain both tumor and non-tumor cells, which include infiltrated immune cells and stromal cells, collectively called the tumor microenvironment (TME). Single-cell RNA sequencing (scRNAseq) enables the examination of heterogeneity of tumor cells and TME. In this review, we examined scRNAseq datasets for multiple cancer types and evaluated the heterogeneity of major cell type composition in different cancer types. We further showed that endothelial cells and fibroblasts/myofibroblasts in different cancer types can be classified into common subtypes, and the subtype composition is clearly associated with cancer characteristic and therapy response.

## Introduction

The hallmarks of cancer consist of complex biological processes include uncontrolled cell growth, induction of angiogenesis, and activation of invasion and metastasis^1^. Cancer cells growing in Petri dishes can only reflect a part of cancer biology. Tumor tissues contain not only tumor cells but also a complex environment supporting tumor cell growth, including blood vessels, infiltrated immune cells, stromal cells, signaling molecules, and extracellular matrix, which is collectively called the tumor microenvironment (TME)^2^. Tumor–TME interactions are important for tumor progression^3^, chemotherapy response^4^, and immune therapy response^5^. Single-cell RNA sequencing (scRNAseq) technology has revolutionized our ability to examine heterogeneity of tumor cells and TME as well as tumor–TME interactions in detail. scRNAseq has been applied to study TMEs in multiple cancer types, including gastric cancer^6^, melanoma^7–9^, uveal melanoma^10^, breast cancer^11,12^, colon cancer^12,13^, hepatocellular carcinoma (HCC)^14^, head and neck squamous cell carcinoma (HNSCC)^15^, lung cancer^12^, ovarian cancer^12^, bladder cancer^16^, and kidney cancer^17^. How tumor-infiltrating immune cells in TME affect tumor progression, and immune therapies has been extensively studied and reviewed^18–20^. In this review, we focus on the heterogeneity of stromal cells in TME.

## A brief history of scRNAseq research

The dramatic decrease in sequencing cost and increase in sequencing throughput around 2010 made it possible to examine individual cells instead of individual tumor, person, or species. Navin et al.^21^ reported sequencing single-cell DNA to elucidate tumor cell evolution in breast cancer progression and metastasis in 2011. Around the same time, multiple groups reported sequencing individual cells at the transcription level, scRNAseq, such as Dalerba et al.^22^ on transcriptional heterogeneity in human colon tumors and Ramskold et al.^23^ on individual circulating tumor cells. The early challenges of scRNAseq studies include how to isolate single cells and how to unbiasedly amplify individual cell’s genome or transcriptome^24^. The early computational challenges include how to cluster and visualize single-cell data^25^ and how to infer missing values in the data^26,27^. Initial applications of scRNAseq focus on discovering novel cell types or cell states. With accumulation of more and more scRNAseq data, the chance of discovering novel cell types/subtypes are diminishing while other challenges arise, e.g., how to integrate different scRNAseq datasets and consistently classify cells into common cell types, how to classify and annotate cells based on information derived from other studies^28^, commonly known as transfer learning^29^.

In this review, we collected multiple solid-cancer scRNAseq datasets consisting of >2,000 cells profiled (**Table 1**). To overcome differences due to data generation platforms (10× Genomics, Smart-seq, etc.), we reprocessed all datasets using a single data-processing pipeline (details in the Methods section), then clustered the cells into major cell types. After the initial clustering step, we collected stromal cells and further classified them into subtypes defined in pan-cancer analyses by Qian et al.^12^ so that we can examine stromal cell heterogeneity across different tumors and adjacent normal tissues.

**Table 1:** Information of the large scRNAseq datasets collected and analyzed in the review

Dataset	Cancer	Platform	No. cell after QC	No. tumor samples	Cells sorted?	No. adjacent normal	No. cell from tumor tissues	No. cell from normal tissues	Age
Sathe et al. (PMID: 32060101)	Gastric cancer	10× Genomics	44,684	8	No	8	30,626	14,058	Yes
GSE115978	Melanoma	Smart-seq2	5,910	31	CD45+ and CD45−	0	5,910	0	Yes
GSE72056	Melanoma	Smart-seq2	3,883	19	CD45+ and CD45−	0	3,883	0	Yes
GSE139829	Uveal melanoma	10× Genomics	116,752	11	No	0	116,752	0	Yes
Qian et al. (PMID: 32561858)	Breast cancer	5′-scRNA-seq	44,024	14	No	0	44,024	0	Yes
Qian et al. (PMID: 32561858)	CRC	10× Genomics	44,684	7	No	7	30,626	14,058	Yes
GSE125449	HCC	10× Genomics	9,581	19	No	0	9,581	0	Yes
GSE103322	HNSCC	Smart-seq2	4,849	18	CD45-, CD45-/CD90-/CD31-, CD45+, CD45+/CD3+	0	4,849	0	No
Qian et al. (PMID: 32561858)	Lung cancer	10× Genomics	93,575	7	No	7	66,309	27,266	Yes
Qian et al. (PMID: 32561858)	Ovarian cancer	10× Genomics	45,114	5	No	2	34,469	10,645	Yes
GSE130001	Bladder cancer	10× Genomics	4,077	2	CD45-	0	4,077	0	No
Young et al. (PMID: 30093597)	Kidney	10× Genomics	72,394	11	No	11	22,476	49,918	Yes

## Major cell types in scRNAseq datasets

**Figure 1:**
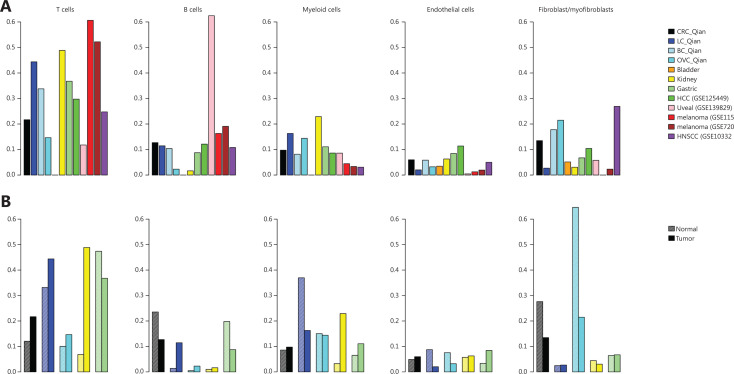
Cell compositions in tumor microenvironment derived from scRNAseq datasets. (A) Fractions of major cell types in different cancer types. (B) Comparison of major cell fractions in different tumor tissues and corresponding adjacent normal tissues.

Cells in each dataset were clustered individually following the procedure described by Qian et al.^12^ and annotated using common cell-specific markers^12,13,16,17^. The resulting cell fractions in TME (summarized in **Figure 1A** and detailed in **Table 2**) were similar to the ones reported in the original studies. For infiltrated immune cells, uveal melanoma had the lowest T-cell infiltration and the highest B-cell infiltration compared with other solid tumor types, which may explain why uveal melanoma had very low response rate to immune checkpoint inhibitors^30^. Meanwhile, kidney tumors had the highest fraction myeloid cells infiltrated into the tumors, which explains why myeloid cells play a profound tumor-promoting role in kidney cancer^31^. Among stromal cells, HCC had the highest endothelial cell (EC) fraction, whereas HNSCC had the highest fibroblast/myofibroblast fraction.

**Table 2:** Cell numbers of major cell types in tumor tissues of different cancer types in the scRNAseq datasets listed in **Table 1**

Cell type	CRC_Qian	LC_Qian	BC_Qian	OVC_Qian	Bladder	Kidney	Gastric	HCC (GSE125449)	Uveal (GSE139829)	melanoma (GSE115978)	melanoma (GSE72056)	HNSCC (GSE103322)
B	3,889	7,590	4,566	790	0	369	2,388	1,160	72,899	963	741	523
DC	0	1,353	166	0	0	0	0	0	0	24	16	0
EC	1,831	1,340	2,568	1,113	142	1,413	2,309	1,089	525	74	75	242
Fibroblast	2,114	0	6,463	6,934	139	124	1,836	157	6,720	0	90	659
Mast cell	440	970	367	0	0	464	295	0	0	0	0	123
Myeloid	2,988	10,805	3,597	4,963	0	5,150	3,022	826	10,030	263	130	150
T	6,635	29,422	14,877	5,055	0	10,981	10,045	2,850	13,763	3,584	2,027	1,200
Alveolar	0	1,465	0	0	0	0	0	0	0	0	0	0
Enteric glia	124	0	0	0	0	0	0	0	0	0	0	0
Epithelial	10,590	11,571	10,056	15,144	3,726	3,412	7,426	2,658	12,815	1,002	804	1,306
Myofibroblast	2,015	1,793	1,364	470	70	563	0	841	0	0	0	646

When comparing cell fractions in tumor tissues and adjacent normal tissues (**Figure 1B, Table 3**), T-cell infiltration was higher in tumor tissues than in the corresponding adjacent normal tissues for all cancers except gastric cancer. The largest difference in T-cell infiltration between tumor and normal tissues was in the kidney. Similarly, kidney tumors had much higher fraction of infiltrated myeloid cells than adjacent normal tissues, indicating the tumor-promoting role of myeloid cells in kidney cancer^31^. It is worth noting that the B-cell fraction in normal colon tissues was much higher than that in tumor tissues, consistent with the role of B cell in response to gut microbiota^32^.

**Table 3:** Cell numbers of major cell types in adjacent normal tissues of different cancer types in the scRNAseq datasets listed in **Table 1**

Cell type	CRC_Qian	LC_Qian	OVC_Qian	Kidney	Gastric
B	3,309	387	53	514	3,623
DC	0	540	0	0	0
EC	688	2,383	806	2,870	615
Fibroblast	3,329	0	5,689	554	1,173
Mast cell	261	230	0	35	97
Myeloid	1,209	10,075	1,599	1,624	1,191
T	1,694	9,046	1,072	3,419	8,672
Alveolar	0	3,555	0	0	0
Enteric glia	691	0	0	0	0
Epithelial	2,324	378	238	39,233	2,937
Myofibroblast	553	672	1,188	1,669	0

## Endothelial cells

**Figure 2:**
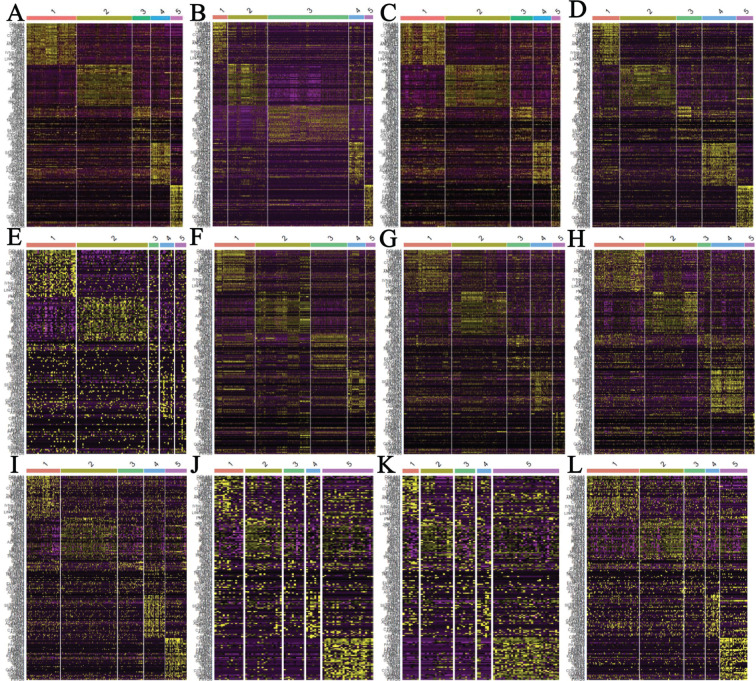
Heatmaps of endothelial cell subtypes in different cancer types. (A) CRC, (B) lung cancer, (C) breast cancer, (D) ovarian cancer, (E) bladder cancer, (F) kidney cancer, (G) gastric cancer, (H) HCC (GSE125449), (I) uveal melanoma (GSE139829), (J) melanoma (GSE115978), (K) melanoma (GSE72056), and (L) HNSCC (GSE103322). The subtype-specific genes were from Qian et al.

Angiogenesis is a key feature of tumor growth. We previously showed that the fraction of ECs in tumor tissues were associated with patient survival^16^, especially in kidney cancers. Based on pan-cancer scRNAseq data analysis, Qian et al.^12^ classified ECs into 5 subtypes: C1_ESM1 for tip cells with high expression of *ESM1*, C2_ACKR1 for venous ECs with high expression of *ACKR1*, C3_CA4 in capillary ECs with high expression of *CA4*, C4_FBLN5 in arterial ECs with high expression of *FBLN5*, and C5_PROX1 lymphatic ECs with high expression of *PROX1*. Qian et al.^12^ also identified 40 EC subtype–specific genes for each subtype. Instead of pooling all ECs in different datasets together and clustering, in which biology differences of different cancers and batch effects are confounded, we leveraged transfer learning approaches to classify EC cells into the 5 subtypes based on the known subtype-specific genes described above, and the resulting heatmaps (**Figure 2**) show clear subtype-specific patterns. Thus, the EC subtype–specific genes were generally applicable across different cancer types in classifying cells profiled using different scRNAseq platforms.

**Figure 3:**
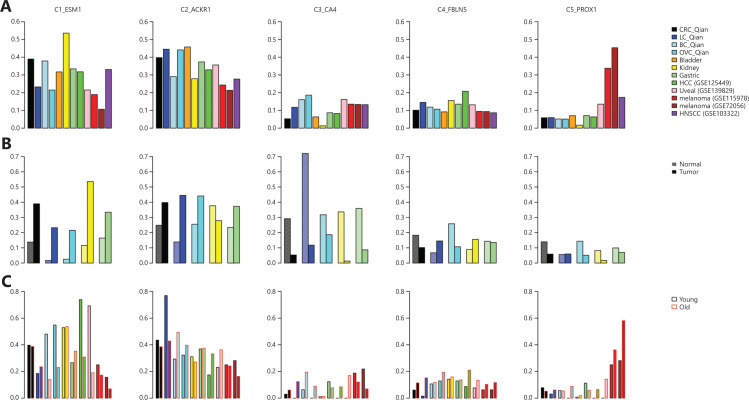
Endothelial cell (ECs) in tumor microenvironment. (A) Fractions of EC subtypes in different cancer types. (B) Comparison of different EC subtype fractions in different tumor tissues and corresponding adjacent normal tissues. (C) Comparison of different EC subtype fractions in tumor tissues from different young and old cancer patients.

The EC subtype frequency in different cancers are shown in **Figure 3A**. Among ECs, kidney tumor tissues contained the highest fraction of tip ECs (C1_ESM1), which is consistent with the fact that kidney cancer responds well to anti-angiogenesis vascular endothelial growth factor (VEGFR) tyrosine kinase inhibitors (TKIs)^33^, whereas melanoma tissues contained the highest lymphatic ECs (C5_PROX1) consistent with prone lymph node metastasis of melanoma^34^.

Compared with adjacent normal tissues, tumor tissues contained consistently higher fractions of tip ECs (C1_ESM1) (**Figure 3B**), suggesting higher angiogenesis activity in tumor tissues, and anti-angiogenesis therapies are used in colorectal (CRC), breast, lung, and ovarian cancers^35–38^. Meanwhile, tumor tissues contained lower fractions of capillary ECs (C3_CA4). The most noticeable difference is that adjacent normal lung tissues contained the highest fraction of capillary ECs (>70% of all ECs), consistent with general lung function.

When patients were split into according to age, young (age <60 years) and old (age ≥60 years) groups, several interesting patterns were revealed (**Figure 3C**). Tumor tissues of young patients with breast, ovarian, liver cancers, and melanoma contained much higher fraction of tip ECs than the corresponding fraction in tumor tissues of old patients. It has been observed that older patients with melanoma respond poorly to anti-VEGFR anti-angiogenesis therapy^39^. The melanoma tissues of older patients contained higher fraction of lymphatic ECs (C5_PROX1) than those of younger patients, consistent with the observation that melanoma in older patients is more aggressive^40^.

## Fibroblasts and myofibroblasts

**Figure 4:**
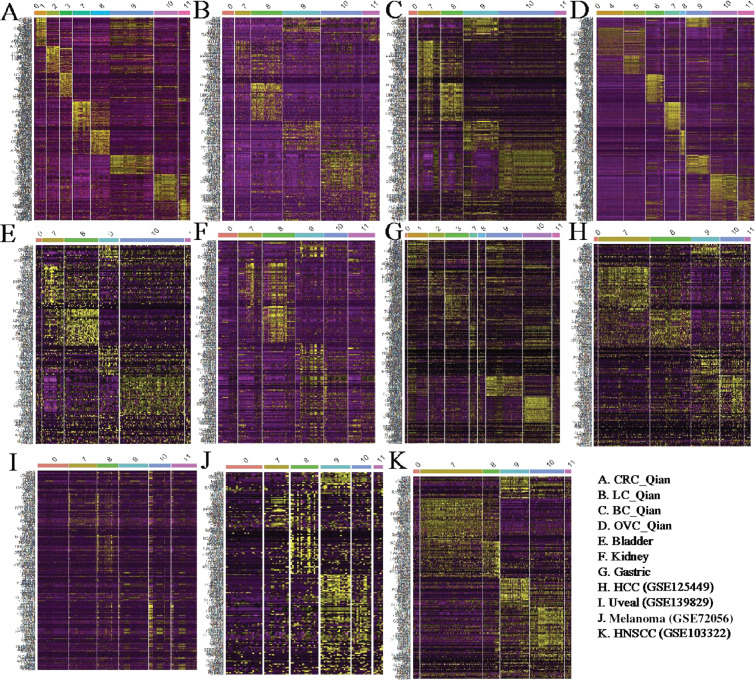
Heatmaps of fibroblast/myofibroblast subtypes in different cancer types. The subtype-specific genes were from Qian et al.

Compared with ECs, fibroblasts are more heterogeneous^41^ and some subtypes are tissue type–specific^12^. When analyzing CRC, ovarian, and lung cancers together, Qian et al.^12^ identified 3 colon tissue–specific fibroblast subtypes, 3 ovary–specific fibroblast subtypes, and 5 fibroblast/myofibroblast subtypes common across all 3 cancer types. In this analysis, we focused on subtypes common across cancer types: C7_MYH11 myofibroblasts with high expression of *MYH11*, C8_RGS5 pericytes involving in angiogenesis and vessel maturation, C9_CFD adipogenic fibroblasts with high expression of adipsin *CFD*, C10_COMP fibroblasts with high activity in transforming growth factor beta (TGF-β) signaling and glycolysis pathways, and C11_SERPINE1 fibroblasts with high expression of genes involved in cell migration and wound healing. Similar to the above analysis of ECs, we collected fibroblasts/myofibroblasts after clustering cells into major cell types. Then, we classified fibroblasts/myofibroblasts into subtypes defined by Qian et al.^12^. Since we encompassed more cancer types in the analysis, there could be tissue-specific fibroblast/myofibroblast subtypes that are not covered by Qian et al.^12^, and we classified these cells as unknown subtypes in the transfer learning process (Methods section). For all the datasets analyzed here, a majority of fibroblasts/myofibroblasts could be classified into 1 of the 5 common fibroblast/myofibroblast subtypes as indicated in the heatmaps of subtype-specific gene expression (**Figure 4**).

**Figure 5:**
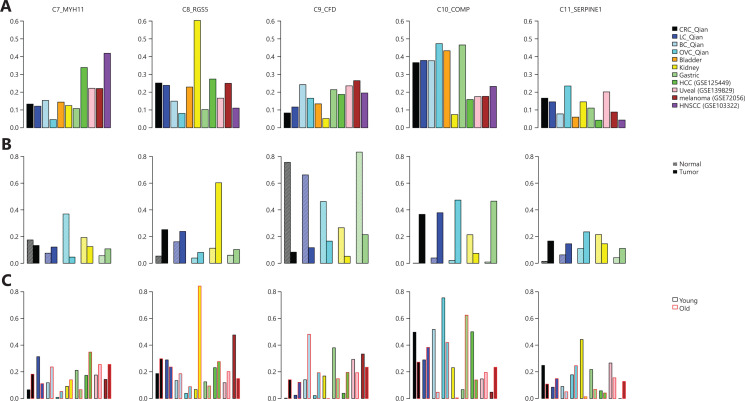
Fibroblasts/myofibroblasts in tumor microenvironment. (A) Fractions of fibroblast/myofibroblast subtypes in different cancer types, (B) Comparison of different fibroblast/myofibroblast subtype fractions in different tumor tissues and corresponding adjacent normal tissues, (C) Comparison of different fibroblast/myofibroblast subtype fractions in tumor tissues from different young and old cancer patients.

Excluding tissue-specific subtypes, we calculated subtype frequencies among the 5 common fibroblast/myofibroblast subtypes in each cancer type (**Figure 5A**). Among all cancer types, HNSCC contained the highest fraction of C7_MYH11 myofibroblasts, consistent with significant roles of myofibroblasts in HNSCC invasion and progression^42^. Liver cancer had the second highest fraction of C7_MYH11 myofibroblast (**Figure 5A**). Liver myofibroblasts, which can be derived from hepatic stellate cells and portal mesenchymal cells^43^, are closely associated with liver fibrosis, liver cancer tumorigenesis, and progression^44^. Compared with other cancer types, kidney cancer had a much higher fraction of C8_RGS5 pericytes (**Figure 5A**). The result, together with a larger fraction of tip ECs in kidney cancer (**Figure 3A**), suggests high angiogenesis activity in kidney cancer^45^. Compared with other cancer types, kidney cancer had the lowest fraction of C10_COMP fibroblasts (**Figure 5A**), which activate TGF-β signaling and glycolysis pathways, but normal kidney tissue had the highest fraction of C10_COMP fibroblasts compared with normal tissues of other origins (**Figure 5B**). In normal renal cells, activation of the TGF-β signaling pathway has protective effects against kidney injury^46^. Similar to the observation that C9_CFD fibroblasts exist mainly in normal colon, lung, and ovary tissues^12^, the fraction of C9_CFD fibroblasts in normal tissue was much higher than that in tumor tissue of the same tissue origin (**Figure 5B**).

When compared with tissues from young patients (**Figure 5C**), tumor tissues from old patients contained a higher fraction of C7_MYH11 myofibroblasts except lung and gastric cancers. It is worth noting that the fraction of C11_SEPINE1 fibroblasts, which are associated with tumor invasion, in tissues of young CRC and gastric cancer patients was higher than that in old patients, consistent with the observation that tumors of young patients with CRC and those with gastric cancer are more invasive^47,48^. Meanwhile, the C11_SEPINE1 fibroblasts fraction in tissues from old patients with melanoma was higher than that in younger patients, suggesting melanoma in older patients is more aggressive^40^.

## Prognosis and chemo-response–related gene signatures in stromal and tumor cells

**Figure 6:**
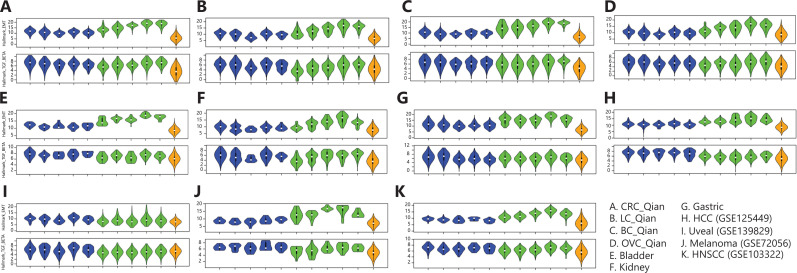
Comparison of the epithelial–mesenchymal transition (EMT) and transforming growth factor beta (TGF-β) signaling pathway activities in different subtypes of stromal cells and cancer cells in tumor tissues. The 2 rows in each panel are activities of the EMT and TGF-β signaling pathways, respectively. Columns represent different cell types. Blue, endothelial subtypes (C1_ESM1 tip ECs, C2_ACKR1 venous ECs, C3_CA4 capillary ECs, C4_FBLN5 arterial ECs, and C5_PROX1 lymphatic ECs); greens, fibroblast/myofibroblast subtypes (C7_MYH11 myofibroblasts, C8_RGS5 pericytes, C9_CFD adipogenic fibroblasts, C10_COMP fibroblasts, and C11_SERPINE1fibroblasts); orange, tumor cells.

**Figure 7:**
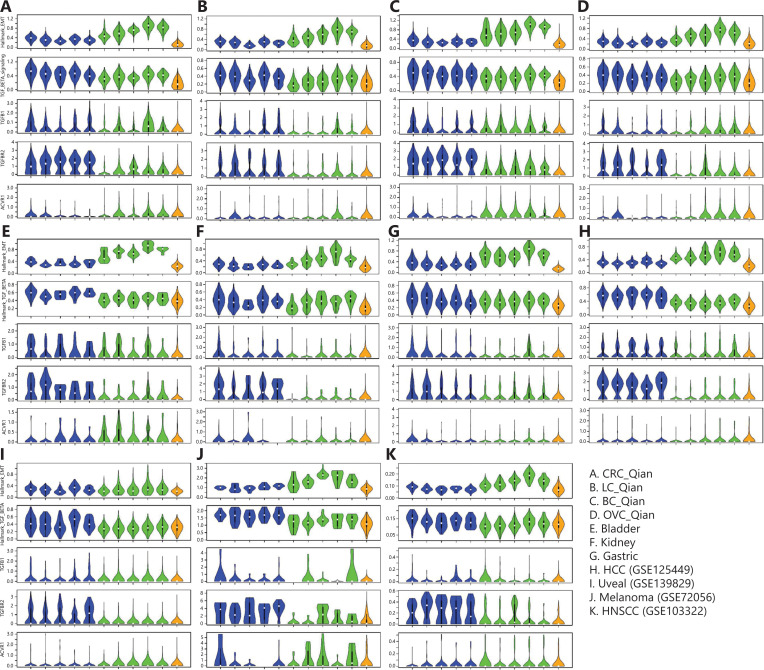
Comparison of expression levels of genes in the epithelial–mesenchymal transition (EMT) and transforming growth factor beta (TGF-β) signaling pathways in different subtypes of stromal and cancer cells in tumor tissues. TGFB1, TGFBR2, and ACVR1 in the TGF-β signaling pathway were also examined individually. Rows in each panel are expression levels of genes in Hallmark_EMT, genes in TGF_BETA_signaling, TGFB1, TGFBR2, and ACVR1, respectively. Blue, endothelial subtypes (C1_ESM1 tip ECs, C2_ACKR1 venous ECs, C3_CA4 capillary ECs, C4_FBLN5 arterial ECs, and C5_PROX1 lymphatic ECs); greens, fibroblast/myofibroblast subtypes (C7_MYH11 myofibroblasts, C8_RGS5 pericytes, C9_CFD adipogenic fibroblasts, C10_COMP fibroblasts, and C11_SERPINE1fibroblasts); orange, tumor cells.

The epithelial–mesenchymal transition (EMT) process confers tumor cell plasticity and is associated with tumor invasion and metastasis and cancer patient survival^49^. Similarly, the TGF-β-signaling pathway activity is associated with tumorigenesis, tumor progression, and cancer patient survival in a more cancer type–specific manner^50^. EMT and TGF-β signaling pathway activities in tumor are also associated with resistance to chemotherapies^51^ and, more recently, to resistance to checkpoint blockade inhibitors as well^52,53^. In tumor tissues, the EMT process and TGF-β signaling pathway activity in tumor cells are not self-regulated; rather, they depend on paracrine signaling from TME^54,55^. With scRNAseq data available in multiple cancer types, we compared expression of genes in EMT and TGF-β signaling pathways in different EC subtypes, fibroblast/myofibroblast subtypes, and tumor cells (**Figure 6**). In all cancer types except uveal melanoma, the EMT and TGF-β signaling pathway activities were higher in stromal cells than in tumor cells (**Figure 6**), and their activities were the highest in cancer-associated fibroblasts (C10_COMP and C11_SERPINE1 fibroblasts), consistent with the observations of Qian et al.^12^. At individual gene level, the expression of genes in the EMT pathway was much higher in fibroblasts/myofibroblasts than in tumor cells (**Figure 7**). Among fibroblast/myofibroblast subtypes, EMT genes expressed at a higher level in C10_COMP and C11_SERPINE1 fibroblast subtypes than in other subtypes (**Figure 7**). For genes in the TGF-β signaling pathway, their expression in both ECs and fibroblasts/myofibroblasts was higher than in tumor cells. These results suggest that the EMT process and TGF-β signaling pathway activity in tumor cells highly depend on paracrine signaling from stromal cells. Among genes in TGF-β signaling pathways, their expression was highly heterogeneous across different genes in the pathway, cancer types, and stromal cell subtypes. The expression of genes within cells of the same subtype in the same cancer type was also heterogeneous. For example, *ACVR1*, a member of the TGF-β signaling pathway, was expressed at a very low level in all ECs, fibroblasts/myofibroblasts, and tumor cells. *TGFBR2*, was expressed at a higher level in all EC subtypes than in fibroblasts/myofibroblasts and tumor cells, and its expression level showed a bimodal distribution in ECs in most cancer types.

## Discussion

In this pan-cancer scRNAseq data analysis, we determined that there was heterogeneity of major cell type compositions in TME of different cancer types. We further demonstrated that the subtype-specific genes of ECs and fibroblasts/myofibroblasts defined by Qian et al.^12^ could be used to robustly classify stromal cells in all cancer types analyzed here (**Figures 3 and 7**). The composition of stromal cell subtypes varied in different cancer types and age groups and was associated with therapy response. For example, kidney tumor contained the highest fraction of tip ECs (C1_ESM1) and responds well to anti-angiogenesis TKIs^33^, whereas melanoma in old patients contained a much lower fraction of tip ECs, and old patients with melanoma respond poorly to anti-VEGFR anti-angiogenesis therapy^39^. We showed that the EMT process in cancer cells highly depended on paracrine signaling in stromal cells (**Figure 6**). Cancer cell lines have very different chemo-sensitivity with and without interaction with stromal cells^56^. As stromal cells are heterogeneous and have disparate effects in interacting with cancer cells and response to anti-cancer drugs, future drug sensitivity screening studies and therapeutic interventions need to consider interactions between cancer cells and different subtypes of stromal cells.

## Methods

### scRNAseq datasets

We collected multiple large scRNAseq datasets with at least 2,000 cells profiled on solid tumors existing in literature including gastric cancer^6^, melanoma^7–9^, uveal melanoma^10^, breast cancer^11,12^, colon cancer^12,13^, HCC^14^, head and neck cancer^15^, lung cancer^12^, ovarian cancer^12^, bladder cancer^16^, and kidney cancer^17^. As we focused on stromal cells in TME, we kept only 12 CD45− or unsorted scRNAseq datasets in our analyses. The description of patient cohorts and information of these datasets are summarized in **Table 1**.

### Data preprocessing

Different datasets were generated using different single-cell RNAseq platforms and analyzed with different pipelines. To reduce biases caused by different processing and analysis methods, we reanalyzed the datasets from raw data with an identical pipeline. For datasets generated using 10× Genomics and other platform with UMI, following the criteria used by Qian et al.^12^, cells with >200 genes and <6,000 genes, with mitochondrial read fraction <25%, and with >400 UMIs were selected for further analyses. Cell cycle score per cell was calculated based on cell cycle genes from Tirosh et al.^8^. The gene expression for each cell was log-normalized with scale factor of 10,000. The top 2,000 most variable genes were selected for clustering analysis based on the variance-stabilizing transformation (VST) method^57^. These genes’ expression was scaled using linear regression to remove effects associated with mitochondrial reads fraction, sample identity, number of UMIs, and cell cycle scores.

For datasets generated using Smart-seq2 and other platform using full transcript, the transcript length–normalized data such as FPKM were transformed to TPM. The log2(TPM/10 + 1) transformation was used as input for further analyses. Following the criteria of Qian et al.^12^, cells with >200 genes and <6,000 genes and with mitochondrial read fraction <25% were selected for further analyses. The cell cycle score for each cell was calculated based on cell cycle genes from Tirosh et al.^8^. Similar to the UMI-based datasets, the top 2,000 most variable genes were selected for clustering analysis using the VST method^57^. The expression data were scaled by mitochondrial reads fraction, sample identity, and cell cycle score.

### Identifying major cell types

After the expression data were scaled, dimension reduction with PCA was performed for each dataset as outlined by Qian et al.^12^. Elbow plot was used to find the optimal number of dimensions for cell clustering. We used common cell type markers^12,13,16,17^ to annotate the resulted clusters. If the reference provides cell type identity, we cross-referenced our annotation results with original published ones. We found that our estimated fractions of major cell types matched well with the original results published in literature.

### EC subtypes

Based on the above major cell type annotation results, we collected the ECs in each dataset. A model for each EC subtype was built based on subtype-specific genes as reported by Qian et al.^12^. Then, each EC was classified by comparing expression of the EC subtype–specific genes in each cell with the 5 EC subtype models one by one. For the UMI-based platform, normalized counts with a scale factor of 10,000 were used as gene expression values. For a full-length transcript-based platform, log2(TPM/10 + 1) was used as gene expression values.

### Fibroblast and myofibroblast subtypes

We collected fibroblasts and myofibroblasts in clustering in each dataset. A model for each fibroblast/myofibroblast subtype was built based on subtype-specific genes reported by Qian et al.^12^. The expression data of the fibroblast/myofibroblast subtype–specific genes in each cell were compared with the 11 tissue-specific and common subtype models. As there could be fibroblast/myofibroblast subtypes beyond the 11 subtypes identified by Qian et al.^12^, we classified a cell as unknown if its expression pattern was not significantly similar to any fibroblast/myofibroblast subtype model. Similar to the classification of EC subtypes, log-normalized counts with scale factor of 10,000 for the UMI-based platform and log2(TPM/10 + 1) for the full-length transcript-based platform were used as gene expression values.

## Pathway activities estimated using single-sample gene set enrichment analysis

The signature gene sets Gene Hallmark_Epithelial_Mesenchymal_Transition (EMT) and HALLMARK_TGF_BETA_SIGNALING (TGFB) were collected from MsigDB^58^. For stromal cells (ECs and fibroblast cells) and tumor cells, we applied single-sample gene set enrichment analysis^59^ version 2.0 based on the 2 signature gene sets and normalized expression. For 10× Genomics and other platforms with UMI, we used log-normalized counts with scale factor of 10,000. For datasets generated using Smart-seq2 and other platforms using full transcript, we used log2(TPM/10 + 1).
